# From Substance Use Disorders in Life to Autopsy Findings: A Combined Case-Record and Medico-Legal Study

**DOI:** 10.3390/ijerph16050801

**Published:** 2019-03-05

**Authors:** Louise Brådvik, Peter Löwenhielm, Arne Frank, Mats Berglund

**Affiliations:** 1 Division of Psychiatry, Department of Clinical Sciences Lund, Faculty of Medicine, Lund University, SE-22185 Lund, Sweden; mats.berglund@med.lu.se; 2 Department of Forensic Medicine, Faculty of Medicine, Lund University, SE-22185 Lund, Sweden; Peter.Lowenhielm@sjobo.nu; 3 Lund University, SE-22185 Lund, Sweden; deceased@gmail.com

**Keywords:** suicide risk, substance use disorders, autopsy findings, case records, depression

## Abstract

**Objectives:** Several studies have shown mortality and suicide risk in substance use disorders, and autopsy findings with respect to the used substances. However, there seems to be a gap in the knowledge about substances misused in life and at death at the within-person level. **Methods:** All consecutive, autopsied patients during 1993 to 1997, who had been in contact with the Addiction Centre in Malmö from 1968, were investigated (365 subjects). Drug misuse in the long-term course noted in case records was related to autopsy findings. Self-inflicted death (suicide/undetermined suicide/accidental overdose) was compared with natural death. **Results:** Benzodiazepine misuse was associated with a high risk of autopsy findings of the substance in suicide and death of undetermined intent. It was also associated among non-misusers, but less so. An alcohol level above 1‰ was found more often in self-inflicted death. Prescription opioids at autopsy were mainly found in self-inflicted death among non-misusers. Heroin misuse was related to overdose. Central nervous system stimulants (CNS-S) and cannabis were rarely found in self-inflicted death among previous misusers. The overlap between depression in life and antidepressants at death was low. **Conclusions:** Benzodiazepines and alcohol seem to disinhibit suicidal tendencies. Suicide risk among users of cannabis and CNS-S may be related to other risk factors than acute use. Implications for suicide prevention are discussed.

## 1. Introduction

Substance use disorders are associated with increased mortality, including self-inflicted death such as suicide, death of undetermined intent, and accidental overdoses by heroin [1,2,3,4]. Substances used and misused have also frequently been found in medico-legal samples.

Among dependent or regular users of illicit opiates, overdose has been found to be the most common cause of increased mortality, and suicide is another major cause of death [5,6]. Premature death, including suicide, has been found among amphetamine users [7,8]. Findings about cannabis use and mortality have been contradictory [9,10]. An increased risk of suicide has been found among cannabis users, but this was explained as markers of psychological and behavioural problems rather than overdose [11]. Increased risk of accidental overdose among users of heroin and cannabis in a criminal justice sample has also been found [12]. In medico-legal studies. heroin has been found to be a main intoxicant among illicit drug misusers in Sweden and Norway, and use has increased [13,14]. In addition to heroin, other intoxicants include amphetamine, tetrahydrocannabinol (THC), benzodiazepines, ethanol [15] and cocaine [16].

According to one study, misuse of benzodiazepines among psychiatric patients could not be related to increased mortality compared to controls with similar diagnoses but no misuse [17]. However, Tiihonen et al. found that use of benzodiazepines was associated with an increased risk of suicide, which did not apply to antidepressants and antipsychotics [18,19]. Benzodiazepines have increasingly been found in autopsy samples [15,20,21] during the past decade, with increasing poly-drug use [22], although these are sometimes seen as incidental findings [23] and only constitute a minor contribution to suicide [24,25]. To our knowledge, the risk of intoxication among users of prescription opioids has only been studied indirectly by comparing prescription rates with fatalities, and some investigators have found corresponding decreased rates with less prescription [26,27,28]. Recent studies have shown increased rates of death with increased prescription [29,30]. One autopsy study reported that fatal intoxications with prescription opioids (buprenorphine, codeine, dextropropoxyphene, fentanyl, methadone, oxycodone, tramadol) were found at increasing rates in Finland [31] and increasing rates of heroin and prescription opioids (including oxycodone) in the US [16].

Alcohol use disorders are associated with an increased risk of completed suicide [3]. From a medico-legal point of view, ethanol appears to predominate among mono-intoxications [24,32] and is also commonly found in addition to other drugs [15]. In fatal intoxications, ethanol appears to predominate among mono-intoxications [24,32].

Substance use disorders are, after depression, the most commonly found diagnoses in suicide victims [33,34]. Although suicide and death of undetermined intent are multifactorial, where diagnoses, psychosocial stressors, somatic disease, etc. are important risk factors, the substance itself contributes to the risk by its physical and psychological effects. This is why misused substances are commonly found at autopsy, and several studies have shown increased risk of mortality among those who misuse. However, there is little knowledge about the link between misuse in life and autopsy findings at the within-person level. In one study, it was found that substances found at autopsy had also been used at index admission [35]. Another study showed that the overlap between histories of help-seeking and substance misuse is not simple: some people with a history of misuse show no evidence of use at the time of death and in autopsies, and some with no record of seeking help show evidence of use at death [36]. Consequently, there is a gap in the knowledge concerning the extent to which people who misuse certain substances in life also use them at self-inflicted death on the within-person level. To address this gap, we have revisited a sample of substance users in Malmö, examining clinical data and autopsy findings.

This study is based on a sample of patients treated at the Addiction Centre in Malmö from 1968 onwards, who died in the period 1993 to 1997, and who were autopsied at the Department of Forensics. A pseudo-prospective design was employed, enabling collection of independent information on these cases, including previous addiction, diagnoses, and causes of death. As this design was time-consuming, extending over several years, we chose to revisit the sample rather than start a new sample. The sample has been presented in a previous study on unnatural death and drugs used in life, concluding that number of substances used in life is related to an increased risk of death of undetermined intent and heroin overdoses, but not suicide [37]. A more recent study on the same sample showed that suicidal communication was more often considered non-serious before death of undetermined intent than before suicide, and that the undetermined group also showed higher levels of alcohol in blood at autopsy [38]. The sample does not reflect the substance use pattern of today, but the physical and psychological effects we intended to study remain the same.

The aim of this study was to compare substances misused in life with autopsy findings on a within-person level, to explore the link between substance use disorder in life and autopsy findings. The study design enables exploration of this link, overlooked by previous studies on substance use disorders in life or autopsy findings only. Unnatural self-inflicted death (suicide, death of undetermined intent, and accidental overdoses) was compared with natural death. Differences in rates may be an indication that the substance had contributed to death. The difference between self-inflicted and natural death for “non-misusers” was also calculated. Risk of suicide, death of undetermined intent, and heroin overdoses during the follow-up by substance used in life were estimated. Violent death by external causes was also described. Background factors, such as number of substances used, psychiatric diagnoses (including depression), and non-fatal suicidal behaviour were considered. Finally, poisoning and violent self-inflicted death were compared, and drugs used at death were described.

## 2. Methods

**Figure 1:**
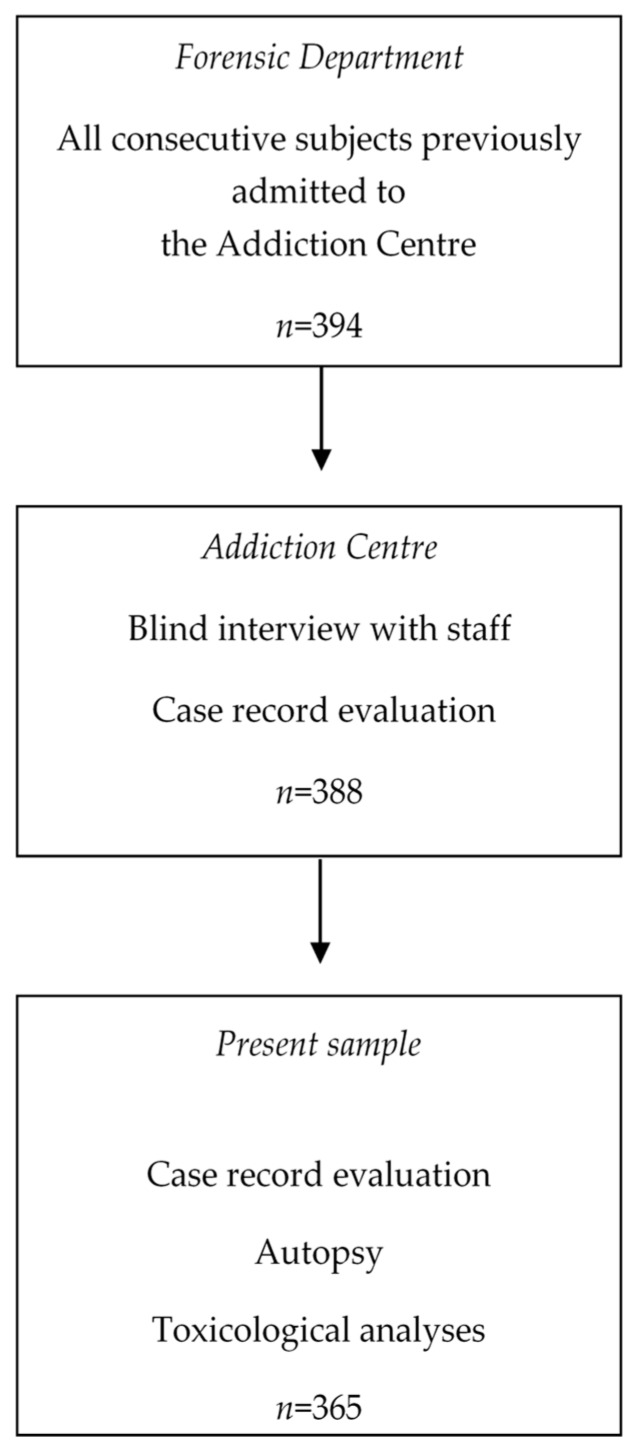
Flow diagram showing the sampling procedure.

All persons autopsied during 1993–1997 at the Forensic Department were matched with the patient register for Addiction Centre in Malmö (originally called Clinical Alcohol Department), which included inpatients and outpatients at the clinic from 1968 and onwards, a follow-up of almost 30 years. Cases with toxicological data were included. The study procedure is presented in Figure 1.

### 2.1. Forensic Examination

A forensic examination sampling procedure was used. This was carried out on all consecutive autopsies of patients who had been in contact with the Addiction Centre at Malmö University Hospital. In Sweden, forensic examination applies to most subjects who have died outside hospitals by suspected natural causes (disease) but with no medical history that can explain the death, or by non-natural manners (trauma including homicide, suicide, death of undetermined intent, and accidental fatal intoxications). Fatal intoxications could be intentional, as in suicide, of unknown intent, as in the case of death of undetermined intent, or probably unintentional, as is usually the case when the opioid drug previously used is involved [39]. The only accidental intoxications in the present sample were heroin overdoses.

Suicide was defined as follows: “Different manners of non-natural death have different numbers of undetermined cases in terms of intent; for example, in a hanging or a shooting it is usually easy to differentiate between a suicide or a trauma (or a crime), while for drowning, traffic accidents or intoxication it is more difficult. Circumstantial findings, such as suicide notes, expressed intent or other findings such as self-inflicted cutting of the wrist followed by drowning, are suggestive of the intent” (E950-953, X60-84-ICD—International Classification of Disease—9 and 10) [40,41]. Death of undetermined intent was defined as follows: “When crime can be ruled out and it cannot be established whether the manner of death is a suicide or an accident, the manner of death is recorded as death of undetermined intent” (E980, Y10-34). Heroin overdose (E850, X40-49) was another cause of self-inflicted death, and it is mostly considered unintentional [6]. The coroner also assessed the contribution of any individual substances to death.

Poisoning and violent (such as hanging, drowning, jumping, burning) methods of self-inflicted death, suicide, and undetermined intent, were noted. Substances found at autopsy, such as benzodiazepines, prescription opioids, and antidepressants, were recorded by manner of death.

Death by trauma, such as fall from height, car accident, and homicide, was considered as violent death (when it was not a suicide). All other cases were considered as natural death, i.e., when the death was caused by disease alone. In some cases, toxicological data were not necessary, for instance some cases of trauma with no suspicion of intoxication.

Mean age at death was 58 years for natural death, 52 for violent, 52 for undetermined intent, 49 for suicide, and 38 for heroin overdoses.

### 2.2. Case Record Evaluations and Interviews

In the period 1993 to 1997 inclusive, 394 consecutive forensic autopsies were performed on previous patients at the Department of Forensic Medicine in Lund. In one case of violent death, it could not be determined whether death was self-inflicted or caused by another person, and case records could not be found for five patients. In 23 cases there was no blood sampling for any substance, so the final sample comprised 365 patients: 319 men and 46 women.

A pseudo-prospective design was used, in which investigation was carried out within a few days of death. One member of the research team (AF) performed the interviews with the staff at the Addiction Centre. As the interviews were performed shortly after death, neither the interviewer nor the interviewees knew the manner of death, whether it was suicide or not. This enabled a blind approach on a reasonable sample size and a reasonable length of follow-up time using the case records. The sampling was carried out in the period 1993–1997, but there have been no significant changes in methodology since then, and the physical and psychological effects of the substances remain the same. The interviewer (AF) blindly evaluated the records of those who had been in- or outpatients at the Addiction Centre in Malmö University Hospital. The items were recorded on a questionnaire, with reports on type and characteristics of the addiction of legal and illegal drugs, physical disease, hallucinations, etc. In this study, information on type of addiction, suicidal behaviour, and diagnoses according to ICD was recorded.

Substance use was diagnosed according to ICD 8 and 9 [40,41] for all inpatients, who constituted 76% of the sample. The remaining 24% had been admitted as outpatients and had applied because they subjectively considered themselves to have a substance use problem and need treatment. It is safe to conclude that almost all outpatients fulfilled the criteria for alcohol dependence and/or had a drug problem. Up to 1994, all the patients treated at the Department of Clinical Alcohol Research were admitted for alcohol problems; after that date, some patients may have been treated for narcotic misuse only, i.e., not for alcohol problems, but in only four cases could alcohol use disorder not be confirmed, though it may still be suspected (1% of the total sample and 4% of the illegal drug users).

Other psychiatric diagnoses according to ICD 8 and 9 [40,41] were also recorded, including misuse of prescribed substances, psychoses and complications to alcohol use disorders. Depression is an important risk factor for suicide, and notes regarding depression and antidepressant medication in the case records were recorded as depression.

Misuse involved both prescribed and illegal drugs. The former was divided into benzodiazepines (and a few cases of “z drugs”) and prescription opioids (dextropropoxyphene and codeine; oxycodone was not misused in Sweden at the time); illegal drugs were divided into opioids, cannabis, and central nervous system stimulants (CNS-S), mainly amphetamine and a few cases of cocaine. All drug misuse was recorded regardless of whether it was the main drug or not. Drugs that are not misused, such as antipsychotics, were not recorded. However, use of antidepressants as a marker of depression was noted.

We present the analysis by drug used in life and found at autopsy. An individual could use more than one drug in life, so more drugs could be found at autopsy. We compared number of drugs used in life and at autopsy for each drug to find out whether poly-drug use was more common for any particular drug as a possible confounder.

### 2.3. Statistics

Fisher’s exact test was used to compare substances used in life and found at autopsy. As there were multiple comparisons, a Bonferroni correction was applied and the significance was set at *p* < 0.0025. A multiple logistic regression was used for comparison between suicidal ideation, suicide attempt, diagnoses, and depression and the different substances used.

Ethical approval was not required for deceased persons in Sweden at that time, and the case records all concerned deceased persons, i.e., none of the sample was still alive. However, the National Board of Forensic Medicine approved the study.

## 3. Results

### 3.1. Case Records: Diagnoses, Suicidal Behaviour, and Misuse

Psychiatric diagnoses and previous non-fatal suicidal behaviour noted in the case records by substance use in life are presented in Table 1. In addition, there were 18 cases of diagnoses of substance use disorder by legal drugs, four of which were not contemporary to another psychiatric diagnosis. Other psychiatric diagnoses tended to be less often reported among cannabis users. Depression was also reported but rarely, in only 20 cases (5–8 per cent for each substance), with no significant difference between the substances used.

Mean number of substances used in life by substances used are presented in Table 2. There were on average around two extra substances, slightly higher for heroin users, and somewhat lower for benzodiazepines.

### 3.2. Autopsy: Manner of Death and Substance Use

Manner of death related to substances used in life is presented in Table 3. Self-inflicted death was more common among patients who had used other substances in addition to alcohol (63–70% versus 31%), mainly because there were no heroin overdoses in the alcohol-only group. The suicide rates were similar for the different substances (around 10%). Death by undetermined intent was common, but somewhat less so among those who had used heroin or alcohol only.

Among those who died by suicide or death by undetermined intent, there were 26 cases of violent method.

### 3.3. Autopsy: Substances Used/Not Misused in Life and Found at Autopsy by Unnatural and Natural Death

Mean number of substances used in life by specific substances used are presented in Table 4. There were similar rates for the different substances used, but the rate was somewhat lower for benzodiazepines. 

**Figure 2:**
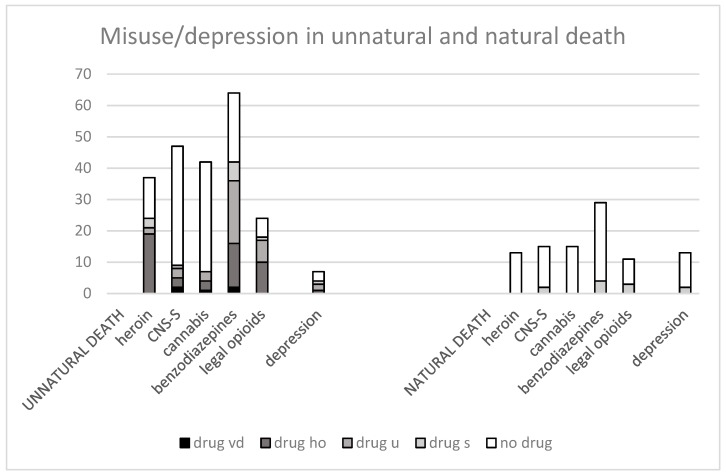
Autopsy findings by manner of death related to substances misused in life and previous depression; violent death (vd) heroin overdose (ho), undetermined intent (u) suicide (s), no drug. Unnatural and natural death. y-axis, number of cases.

Substances used in life related to autopsy findings by manner of death (unnatural or natural death) are presented in Figure 2.

Heroin at autopsy among heroin users was significantly more often found in self-inflicted death compared with natural death (Fisher’s exact test: 23/30 vs. 0/13 *p* < 0.0001). Prescription opioids were also marginally significantly more often found in self-inflicted death (Fisher’s exact test: 18/24 vs. 3/9 *p* < 0.0441). The same association was found for benzodiazepines (Fisher’s exact test: 40/57 vs. 4/29 *p* < 0.0001). When self-inflicted death by heroin overdoses was excluded, use of benzodiazepines was the only substance that remained associated (Fisher’s exact test: 26/40 vs. 4/29 *p* < 0.0001).

In contrast, CNS-S and cannabis were rarely found at autopsy among those who had used the substances in life, regardless of manner of death. No correlations could be shown.

Among those who had used alcohol only, no significantly higher rates of alcohol use were found at autopsy in self-inflicted than natural death (63/148 (43%) vs. 52/156 (33%)). However, there were higher rates of alcohol level above 1‰ among those who died of self-inflicted death (Fisher’s 11/49 (22%) vs. 34/58 (58%) *p* < 0.0002).

**Figure 3:**
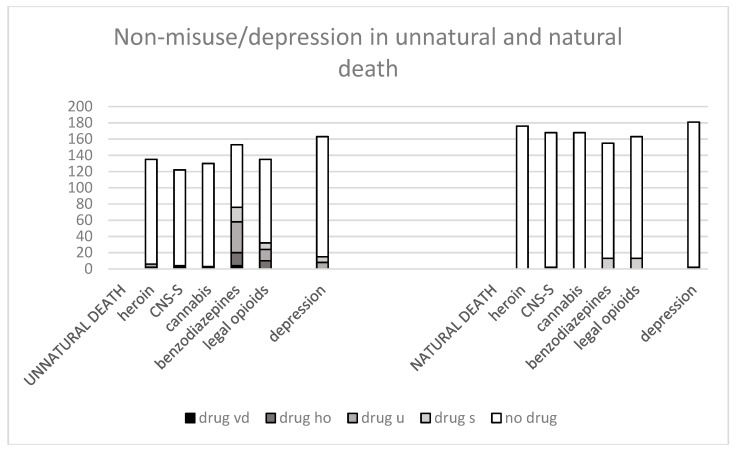
Autopsy findings by manner of death among non-misusers and no previous depression; violent death (vd) heroin overdose (ho), undetermined intent (u) suicide (s), no drug. Unnatural and natural death. y-axis: number of cases.

A comparison between substances found at autopsy by manner of death (self-inflicted or natural death) among non-misusers in life is shown in Figure 3.

Illegal substances were rarely found, regardless of manner of death.

Prescription opioids were more often found at autopsy in self-inflicted deaths compared with natural death among those who had not used the substance in life (Fisher’s exact test; 38/129 vs. 16/201, *p* < 0.0001). If heroin overdoses were excluded, the correlation remained significant (Fisher’s exact test: 16/201 vs. 27/118, *p* < 0.0003).

Benzodiazepines were significantly more often found at autopsy in self-inflicted deaths compared with natural death even among those who did not have a known use/misuse during life (Fisher’s exact test: 32/96 vs. 13/165, *p* < 0.0001). If heroin overdoses were excluded, the result remained significant (Fisher’s exact test: 30/91 vs. 13/165 *p* < 0.0001).

However, the rates were significantly higher for users than for non-misusers (Fisher’s exact test: 26/40 vs. 30/91 *p* < 0.0010).

### 3.4. Autopsy: Substances Detected and Violent Manner of Self-Inflicted Death

Alcohol intoxication was found in similar rates in violent and poisoning death (suicide and death of undetermined intent) (Fisher’s exact test 15/26 vs. 69/105, *p* < 0.4966.) Similar rates of alcohol levels above 1‰ were found in both types of death (Fisher’s exact test: 6/15 vs. 15/69; *p* < 0.1875).

Prescription opioids at autopsy were related to poisoning in self-inflicted death (Fisher’s exact test: 33/105 vs. 1/26 *p* < 0.0025).

### 3.5. Autopsy: Violent Death

Eighteen patients died by violent manner of death. Twelve were positive for high levels of alcohol; mean 3.08‰ (range 2.3–4.6‰). There was one case of benzodiazepine, one of cannabis, and four of CNS-S, but none of opioids. Only two were not positive for any drug. There were two cases of homicide, one positive for alcohol (3.4‰). Intoxication was therefore very common, mainly with respect to alcohol.

### 3.6. Autopsy: Contribution to Death by Substances Found

The contribution of the substances found at autopsy were investigated. There were 56 cases of benzodiazepine found at autopsy in suicide and undetermined intent (30 among previous users), of which there was one case (2%) of major cause of death, 29 cases (52%) of contribution, and 26 cases (46%) of occasional findings, including z-drugs.

For 34 cases of intoxication of prescription opioids in suicide and undetermined intent, there were 15 (44%) main causes of death, 12 (35%) contributions and 7 (21%) occasional. Twenty-four had used dextropropoxyphene; others had used mainly codeine.

Among those who had used cannabis in life, eight had died by suicide by intoxication, but none had been using the drug at death. Three out of 14 who died by undetermined intent had used cannabis, and three out of 16 who died by heroin overdoses. Other intoxicants used were prescription opioids, ethanol, benzodiazepines, venlafaxin, paracetamol, and carbon monoxide.

Among those who had used CNS-S, seven had died by suicide, one of whom had used amphetamine. Four out of 18 who died by undetermined intent had used CNS-S. Other stimulants used were ethanol, opioids, ketamine, paracetamol, karbamazepin, karisopropodol, teophyllin, and orphenadrin.

Among those who had used heroin in life, four had died by suicide, three of whom had been using the drug at death. Seven had died by undetermined intent, one of whom had used heroin. One of those had possibly used heroin before death. They had also used ethanol, prescription opioids, benzodiazepines, karisopropodol, venlafaxin, orfenadrin, and karbamazepin.

### 3.7. Case and: Depression and Antidepressants

There was only one case of overlap between depression detected in life and antidepressants found at autopsy. Of the 20 patients with signs of depression according to case records, seven died by their own hand (35%) versus 146/181 (45%) not diagnosed with depression, thus no significantly higher suicide rate for depressives. Twenty-two positive cases of antidepressants were found at autopsy. Of these 18 died by their own hand vs. 134/324 among those negative (Fisher’s exact test; *p* < 0.0002). In four cases, antidepressants were considered the main cause of death, seven contributing together with other substances, and seven occasional, so antidepressants were considered as cause of death in more than half the cases (61%). Lithium was found in one case of death of undetermined intent.

## 4. Discussion

### 4.1. Main Findings

This study examines the link between substances used in life and autopsy findings on the within-person level.

Benzodiazepine misuse was associated with self-inflicted death. It was also associated with self-inflicted death among non-misusers, but less so. For prescription opioids, there was a significant association with self-inflicted death among non-misusers, regardless of the inclusion of heroin overdoses. Heroin use was, as expected, associated with a risk of overdose. Cannabis and CNS-S were rarely found in self-inflicted death. Alcohol levels above 1‰ were associated with self-inflicted death, but there was no significant difference between poisoning or violent method. Prescription opioids were negatively associated with violent method. High levels of alcohol were often found in violent manner of death.

According to a recent review of 17 studies, a positive correlation between prescription benzodiazepines and non-fatal and fatal suicidal behaviour was reported in most studies, with few exceptions [42]. This has since been questioned [43]. Epidemiological, clinical, laboratory-based, and neuro-biological studies were included. Possible mechanisms of pro-suicidal effects could be an increase in impulsivity or aggression. The reviewed articles included the two above-mentioned longitudinal studies [28,29], where there was an increased risk of suicide in people with schizophrenia, who were prescribed benzodiazepines, while antipsychotics reduced the risk in both studies and antidepressants in the latter. Other studies in the review reported prescription of benzodiazepines shortly before suicide. The present study gives further support for this view, by adding autopsy findings of benzodiazepines among previous users, thereby indicating that the substance was also used at the time of death. There was no indication of specific comorbidity to explain the increased risk, but there were only two cases of anxiety diagnoses (one with misuse of benzodiazepine), which may be an underestimate. We cannot definitely rule out self-medication at the time of suicide, nor that the findings represent withdrawal symptoms.

Alcohol at levels above 1‰ was also associated with an increased risk of self-inflicted death. This level of alcohol has also been found in suicide victims with a brittle/sensitive personality [44] and high levels of alcohol were also related to suicide among patients with suicidal ideation in a previous study on the present sample [38], both indicating disinhibition of suicidal impulses. Furthermore, there were equal rates of positive blood sampling in poisoning and violent suicides and deaths of undetermined intent and also similar rates of detection of high alcohol levels (above 1‰), regardless of violent method. This agrees with a study by Jones et al. [45] showing no significant difference in detection of alcohol in poisoning and hanging suicides. A possible interpretation is that alcohol is compatible with, or even induces, fatal suicidal acts, regardless of the toxic effect.

The association between heroin use and fatal heroin overdoses was expected and has repeatedly been shown in previous literature [5,6]. Prescription opioids were sometimes used in addition to heroin in heroin overdoses. They were also found at autopsy at higher rates in comparison with natural death among non-misusers. When poisoning was compared with violent suicide/death of undetermined intent, prescription opioids were associated with poisoning rather than violent death. These findings may indicate that the toxicity is the main reason of the fatal outcome rather than psychological effects, such as aggression and depression, in agreement with the known effects of opioids. Regular use was not shown to be an increased risk. Dextropropoxiphene was available at the time, which probably increased the risk of self-inflicted death. Reduced suicide rates were shown after withdrawal of this drug from the market [21].

Cannabis and CNS-S were rarely found at autopsy, even in previous users, so acute use could not be associated with self-inflicted death among persons who had previously used these substances. In a recent review [46], it was concluded that there is lack of evidence that acute use of cannabis increases the imminent risk of suicidality, while evidence tends to support that chronic acute use can predict suicide. One study [11] concluded that suicide rates have been found to be increased among cannabis users, but they were explained as markers of psychological and behavioural problems rather than overdose. The present study supports this view. One study showed that there was a smaller number of deaths with amphetamines and cocaine compared to opioids, in agreement with the present findings [4]. However, that study dealt with autopsy findings and did not consider risk among previous users.

The overlap between depression detected in life and antidepressants found at autopsy was remarkably low, only one case. The medication was also considered to contribute to death in more than half of the cases. Depression in alcohol use disorder is probably undertreated and difficult to treat. Men with depression and alcohol use disorder have been shown to have a very high risk of suicide, 16.2% [47]. Furthermore, in comorbid cases of depression and alcohol use disorders, alcohol turned out to be more common as first diagnosis in the same sample [48]. This means that detection and treatment of depression secondary to alcohol are important in the efforts to prevent suicide.

In summary, there was a high risk of self-inflicted death for all the substances, regardless of the acute effect. There was no obvious difference in psychiatric comorbidity or poly-drug use between the substances, apart from fewer diagnoses among cannabis users. However, the latter did not show lower rates of self-inflicted death. The chronic effect on the risk of suicide and death of undetermined intent needs to be considered and investigated. There was a modest overlap between substances used in life and autopsy findings, in agreement with the study on detection of alcohol at autopsy and alcohol use reported in primary care [36]. Another study reported that the same substances used at index admission could be found at autopsy. but did not consider to what extent the substances were found [35]. Substance use as such is a risk factor for suicide, where the use of the substance itself contributes, but the contribution may vary for different substances, as has been clarified in the present study.

Gamma-amino butyric acid (GABA-ergic) substances, such as benzodiazepines and alcohol, seemed to facilitate suicidal behaviour on a psychological level, although it may be difficult to distinguish between physical and psychological effects. The fact that alcohol was also common in violent suicide or undetermined death is compatible with a psychological effect as well, as the physical effect did not contribute to death. Opioids, legal or illegal, appear to mainly have a physical effect, as prescription opioids were associated with suicide in casual use and negatively associated with violent suicide or death of undetermined intent.

Cannabis may have a very limited toxic effect on suicidal behaviour, and social factors may contribute to the suicide risk. CNS-S do not often facilitate suicidal feelings, but aggression may lead to violent behaviour [49]. The number was too small to show an association with violent suicide in the present sample.

The almost non-existent overlap between depression in life and antidepressants at autopsy may be an indication of low detection and undertreatment of depression.

To prevent suicide in substance use disorder, treatment of the misuse itself, and poly-drug use, is of major importance. Benzodiazepines should be prescribed with caution to people with substance misuse. Investigation of suicidal ideation during drug use would be of major importance, as the substance may facilitate suicidal behaviour. Reduced availability of more toxic opioids has already been found to reduce suicide rates [21]. Psychosocial factors should not be overlooked. Comorbidity with depression needs investigation, and treatment of depression in alcohol use disorders, although presenting a challenge, is very important. The association between cannabis and schizophrenia [50] is another possible link to suicidal behaviour.

### 4.2. Strengths and Limitations

The major strength of this study was the combination of clinical data with a long-term follow-up and autopsy findings on a within-person level, an approach that, to the best of our knowledge, has very rarely been used. The design enabled an exploration of the link between the use of substances in life and autopsy findings. The research assistant who evaluated the case records did not know the cause of death, so the assessments were unbiased in terms of knowledge of the manner of death, a problem usually inherent in a retrospective design. The case records have been shown to be of good standard by validation against self-reports [51].

In this study, natural death was restricted to those who were autopsied. Some people treated at the Addiction Centre had died at the hospital. Those patients were either not autopsied at the Forensic Department or not at all, so the risk of self-inflicted death is overestimated. However, those who had died at the hospital could not be assumed to be more often positive for the different substances compared to those who had been autopsied, so the higher doses found in self-inflicted death is probably reliable.

The substances used are not representative of substances used today. However, the physical and psychological effects are the same, and these effects were the aim of this investigation. Furthermore, the increasing poly-drug use today makes it even more difficult to study the effect of individual substances. We could not control for the effect of use of more than one drug, but the number of drugs was similar. This sample is not representative of users of benzodiazepines and prescription opioids without using other substances, mainly alcohol, as well.

Additional psychiatric diagnoses may be underestimated, but this may be equally applicable to the variables compared.

Blood-sampling of drugs and not brain autopsy was used, so we have only information on the recent intake of substances before death.

## 5. Conclusions

This study supports the view that a psychological effect facilitating suicidal behaviour, in addition to a medium toxic effect, contributes to the high risk of self-inflicted death patients who use the GABA-ergic substances benzodiazepine and alcohol. The latter substance was also commonly found in natural death, but in less toxic doses. Legal and illegal opioids, not unexpectedly, appear mainly to have a toxic effect, as accidental overdoses were common and violent death very rare. Use of illegal drugs, such as cannabis and CNS-S, shows a high risk of self-inflicted death from accidental overdoses to suicide, but the chronic risk appears more important than the acute use of the drugs. More knowledge about comorbidity with other diagnoses appears to be an important topic for future research. Detection and treatment of depression in substance use disorders appears to be a challenge.

Caution should be exercised in prescribing benzodiazepines and opioids to people with an alcohol problem. Inquiry about suicidal tendencies and also a history of suicide attempts in patients with substance use disorders, also during intoxicated states, may be important in the efforts to prevent suicide. Finally, people with substance use disorders who are not registered at the hospital should be a topic for future research.

## Figures and Tables

**Table 1:** Type of misuse related to suicidal behaviour and diagnoses in case records.

Misuse	Suicidal Ideation	Suicide Attempt	Diagnoses	Depression
Heroin (*n* = 49)	8 (16)	18 (37)	17 (35)	3 (6)
CNS-S (*n* = 63)	12 (19)	23 (37)	22 (35)	5 (8)
Cannabis (*n* = 56)	7 (13)	18 (32)	14 (25) *	4 (7)
Benzodiazepines (*n* = 91)	27 (30)	33 (36)	40 (44)	5 (5)
Legal opioids (*n* = 35)	11 (31)	15 (43)	16 (46)	2 (6)
Alcohol only (*n* = 231)	35 (15)	29 (13)	76 (33)	11 (5)
All; including alcohol only (*N* = 365)	70 (19)	73 (20)	130 (36)	20 (5)

* Diagnoses cannabis versus other drugs: *p* < 0.008 (multiple regression).

**Table 2:** Mean number of drugs misused in life by each drug used in life. Alcohol excluded.

Substance	Mean Number of Drugs Used in Life
Heroin	3.51 (±0.982)
Central stimulants	3.17 (±1.129)
Cannabis	3.13 (±1.237)
Benzodiazepines	2.34 (±1.400)
Legal opioids	2.91 (±1.463)
All; including alcohol only	0.80 (±1.314)

**Table 3:** Manner of death by substance misused in life in addition to alcohol. (There is an overlap of substances, so the sum does not correspond to individuals.). CNS-S: central nervous system stimulants.

	Heroin	CNS-S	Cannabis	Benzodiazepines	Legal Opioids	Alcohol Only
Natural death *n* = 194 (53%)	13 (27%)	15 (24%)	15 (20%)	29 (32%)	9 (26%)	150 (65%)
Violent death *n* = 18 (5%)	6 (12%)	4 (6%)	3 (5%)	5 (6%)	2 (6%)	10 (4%)
Suicide *n* = 44 (12%)	4 (8%)	7 (11%)	8 (14%)	10 (11%)	2 (6%)	27 (12%)
Death of undetermined intent *n* = 87 (24%)	7 (14%)	18 (29%)	14 (25%)	30 (33%)	11 (31%)	44 (19%)
Heroin overdose *n* = 22 (6%)	19 (39%)	19 (30%)	16 (29%)	17 (19%)	11 (31%)	0 (0%)
Total self-inflicted death *n* = 153 (42%)	31 (63%)	44 (70%)	39 (70%)	57 (63%)	24 (69%)	71 (31%)
Total *n* = 365 (100%)	49 (100%)	63 (100%)	56 (100%)	91 (100%)	35 (100%)	231 (100%)

**Table 4:** Mean number of drugs found at autopsy by drug used in life.

Substances Used/Misused in Life	Mean Number of Drugs Found at Autopsy
Heroin	2.31 (±1.262)
CNS-S	2.29 (±1.197)
Cannabis	2.20 (±1.182)
Benzodiazepines	1.97 (±1.251)
Prescription opioids	2.49 (±1.197)
Alcohol only	1.12 (±0.879)
All; including alcohol only	1.43 (±1.094)
